# A Shared Decision-Making Tool to Prevent Substance Abuse: Protocol for a Randomized Controlled Trial

**DOI:** 10.2196/resprot.7650

**Published:** 2018-01-11

**Authors:** Ju Long, Juntao Michael Yuan, Ron Kim Johnson

**Affiliations:** ^1^ Department of Computer Information Systems and Quantitative Methods McCoy College of Business Texas State University San Marcos, TX United States; ^2^ Ringful Health, LLC Austin, TX United States

**Keywords:** SBIRT, substance abuse, SUD, primary care brief intervention

## Abstract

**Background:**

Substance use disorder (SUD) affects over 20 million adults and costs over $700 billion annually in the United States. It is one the greatest health care challenges we face.

**Objective:**

This research project seeks to enhance the standard practice of Screening, Brief Intervention, and Referral to Treatment (SBIRT) through a mobile solution easily incorporated into primary care that will promote shared decision making and increase referral and adherence to specialty care through continued follow-up care.

**Methods:**

This research will conduct an Office of Management and Budget (OMB)–approved randomized controlled trial (RCT) in primary care and SUD specialty service providers. The RCT will recruit a total of 500 SUD patients. Recruited patients will be randomized into control and intervention arms. Both arms will take initial baseline and exit (30 days) surveys to evaluate self-reported substance use and specialty service utilization. The control arm patients will receive usual care. The intervention group patients will receive technology-enhanced SBIRT and a mobile follow-up program to track goals and substance use at home. The RCT tracks participants for 30 days after the primary care encounter. We will collect feedback from the patients during the 30 days and count the number of patients who use specialty care services in specialty care programs for tobacco, alcohol, and drug abuse (both from self-reporting and from the service providers).

**Results:**

RCT and data collection are underway. We expect to report the data results in 2018.

**Conclusions:**

We expect that significantly more intervention group patients will receive specialty SUD care within 30 days following the SBIRT encounter at the primary care clinic compared to the control group. We also expect that the intervention group patients will report a greater reduction in substance use and a greater drop in Drug Abuse Screening Test and Addition Severity Index scores within 30 days.

## Introduction

### Background

Substance abuse is a serious public health concern in the United States, with severe medical, legal, and economic consequences. In 2012, an estimated 23.9 million Americans aged 12 years or older had used an illicit drug or abused a psychotherapeutic medication (such as a pain reliever, stimulant, or tranquilizer) in the past month, and 17.7 million Americans were dependent on or abused alcohol [1]. Recently, there has been a dramatic increase in the misuse of prescription medications, with emergency department visits involving their abuse increasing by 98.4% between 2004 and 2009 and associated increases in overdose deaths [2]. Abuse of alcohol and illicit drugs is costly, with over $30 billion in health care–related costs for alcohol abuse and $11 billion for illicit drugs [3]. Unfortunately, few needing treatment for drug or alcohol abuse get the treatment they need in a timely manner. According to the Substance Abuse and Mental Health Services Administration’s National Survey on Drug Use and Health, 23.5 million people aged 12 years or older needed treatment for an illicit drug or alcohol abuse problem in 2009, but of these, only 2.6 million received treatment at a specialty facility [4].

### Substance Abuse Screening and Treatment in Primary Care

The health care landscape for patients with substance use disorders (SUDs) is changing. Recently, there has been a push to incorporate mental and behavioral health treatment into primary care, including screening and treatment for alcohol and drug dependence. The Patient Protection and Affordable Care Act, for example, designates mental health and substance use disorders as essential health benefits to be covered by health insurance plans [5]. The US Preventive Services Task Force (USPSTF) recommends screening for alcohol misuse for adults aged 18 years and older and has found sufficient evidence to suggest that brief behavioral counseling interventions in the primary care setting are effective in reducing heavy drinking episodes in adults engaging in risky drinking behaviors [6]. However, the USPSTF has not found sufficient evidence to assess whether screening for illicit drug use in the primary care setting is beneficial, primarily because the majority of studies have focused on patients already exhibiting clear symptoms of drug abuse [7]. Nevertheless, drug-specific pharmacotherapy and behavioral interventions such as brief motivational counseling for illicit drug use have been proven effective in the short-term [7], and the Centers for Medicare and Medicaid Services provides reimbursement to providers who implement the Substance Abuse and Mental Health Services Administration Screening, Brief Intervention, and Referral to Treatment (SBIRT) model in their practices [8]. The SBIRT model is an evidence-based practice designed to identify, reduce, and prevent problematic use, abuse, and dependence on alcohol and illicit drugs and can be incorporated into a primary care practice with the addition of screening questions into the health history questionnaire and training for health care providers to review the responses and identify high-risk patients.

The implementation of screening and intervention for substance use disorders, such as SBIRT, in the primary care setting has faced several challenges. In the past, physicians have cited barriers such as lack of time, lack of access to treatment, and lack of financial resources—both patient financial issues and reimbursement for the physician from health insurers [9]. The new health care changes will do a great deal in easing some of the cost-related barriers; however, physicians and other health care providers still experience barriers to screening and intervention for SUDs such as lack of time, lack of training, and unfamiliarity with screening tools [10]. Implementation of the SBIRT model has been met with similar concerns, with providers citing lack of time and competing medical priorities in the patient interview as barriers to its use [11].

### Shared Decision Making, Self-Monitoring, and Ecological Momentary Assessment

As the fields of mental health care and substance abuse treatment have changed, there has been a greater emphasis on the importance of patient autonomy and patient involvement in treatment. Active patient participation is a critical component of recovery and enhances the personal meaning, treatment satisfaction, and quality of life for the patient [12]. Shared decision making (SDM) is an interactive collaborative process between the health care provider and the patient in which the practitioner becomes a consultant to the patient, providing information, discussing options, and supporting the patient’s autonomy as they mutually decide on treatment options [13]. SDM has been found to be associated with improved outcomes in substance-dependent patients, including increased personal control and reduced drug use [14-15].

One important component of SUD treatment and prevention that contributes to patient autonomy is the use of self-monitoring logs, in which patients are directed to record details about their alcohol and drug use, including their moods and the situations in which use occurred. This practice serves multiple purposes—it provides an opportunity for the patients to talk openly and honestly about their alcohol and drug use, it encourages patients to take responsibility for their own behavior change, and it provides information that providers can use to observe patterns and give feedback about changes in alcohol and drug use over time [16]. Ecological momentary assessment (EMA) is a method in which data are collected in real-world environments as subjects go about their lives. Assessments focus on subjects’ current state based on strategically selected moments (eg, occasions when subjects have craving). In EMA, subjects complete multiple assessments over time, providing a picture of how their experiences and behavior vary over time and across situations [17].

The self-monitoring aspect of SUD prevention and treatment lends itself perfectly to EMA, as EMA can minimize recall bias and provide a clear picture of the patterns involved in substance use [18]. Indeed, EMA has been used successfully in the field of substance use research. EMA methods have helped highlight the processes that drive drug use, cessation, and relapse and provide detailed information on mood variations and their relation to substance abuse that is not possible with other data collection methods [19]. Researchers have shown that EMA is a feasible method for collecting data from methamphetamine-dependent outpatients [20], with recovering alcoholics [21], and with cocaine users [22].

### Technological Approaches to Substance Abuse Disorder Prevention, Screening, and Treatment

In an effort to overcome some of the barriers to screening and prevention of substance use disorders, many researchers have begun to explore novel approaches using Web-based and mobile technology. These mobile technologies often focus on self-help and self-monitoring as an adjunct for traditional, face-to-face treatment in a clinician office setting and in doing so, reduce the time and cost burden on the health care provider.

While the quality of evidence is often inconsistent, there is promising research to indicate that interventions using Web-based or mobile technology for alcohol and other substance abuse can be effective [23-26]. Most recently, brief intervention applications related to substance use have focused on young adult populations and risky drinking behavior. While some studies have shown potential in reducing drinking outcomes [27-28], more research is necessary to determine overall effectiveness and whether the same strategies could be effective for substances other than alcohol. Features such as tailored feedback have shown to be more effective than similar programs without feedback [29], and interventions that combine self-administered therapy in conjunction with therapist-directed interventions show greater reductions in addictive behavior [30]. Future research is needed to determine to what extent such applications can help the primary care physician integrate SBIRT and other substance use screening models into their practice while providing significant outcomes in patient behavior.

## Methods

### Objectives

The main objective of this research will be to conduct an Office of Management and Budget (OMB)-approved randomized controlled trial (RCT) in primary care and SUD specialty service providers affiliated with Washington State University. The RCT will recruit a total of 500 SUD patients from University Health District (UHD) Clinic, a primary care clinic affiliated with the university. The patients will be randomized to receive usual SBIRT care and technology-enhanced SBIRT care. High-risk SUD patients will be referred to university-affiliated specialty providers in the area.

### Hypotheses

The RCT will address 2 primary hypotheses. Hypothesis 1 has 7 secondary hypotheses. Compared with the control group,

1. Significantly more intervention group patients will receive specialty SUD care within 30 days following the SBIRT encounter at the primary care clinic. The secondary hypotheses attempt to explain how and where the increased specialty care utilization occurs in the process.
  - More intervention group patients are diagnosed with high-risk SUD.
  - More intervention group patients are subsequently referred to specialty care at the end of the primary care clinic encounter.
  - More intervention group patients are referred to specialty care during the 30-day following period due to failing to adhere to goals.
  - Intervention group patients are more satisfied with the care they receive.
  - Intervention group patients are more knowledgeable about SUD.
  - Intervention group clinicians are more satisfied with the process.
  - Intervention group clinicians spend less time on the SBIRT intervention and follow-up.
2. Intervention group patients will report a greater reduction in substance use and a greater drop in Drug Abuse Screening Test (DAST-10) and Addiction Severity Index (ASI) scores within 30 days.

### Trial Process

#### Timing and Flow of the Randomized Controlled Trial

We will conduct an RCT with 500 patients with SUD risk in the UHD Clinic, a large academic primary care provider affiliated with a major public university. Recruited patients will be randomized into control and intervention arms. Both arms will take initial baseline and exit (30 days) surveys to evaluate self-reported substance use and specialty service utilization. The control arm patients will receive usual care. The intervention group patients will receive technology-enhanced SBIRT with a mobile follow-up program to track goals and substance use at home. The RCT will be managed by a team of seasoned SBIRT and addiction researchers and practitioners at the research site.

The RCT tracks participants for 30 days after the primary care encounter. We will collect feedback from the patients during the 30 days and count the number of patients who use specialty care services in specialty care programs for tobacco, alcohol, and drug abuse (both from self-reporting and from the service providers).

**Figure 1:**
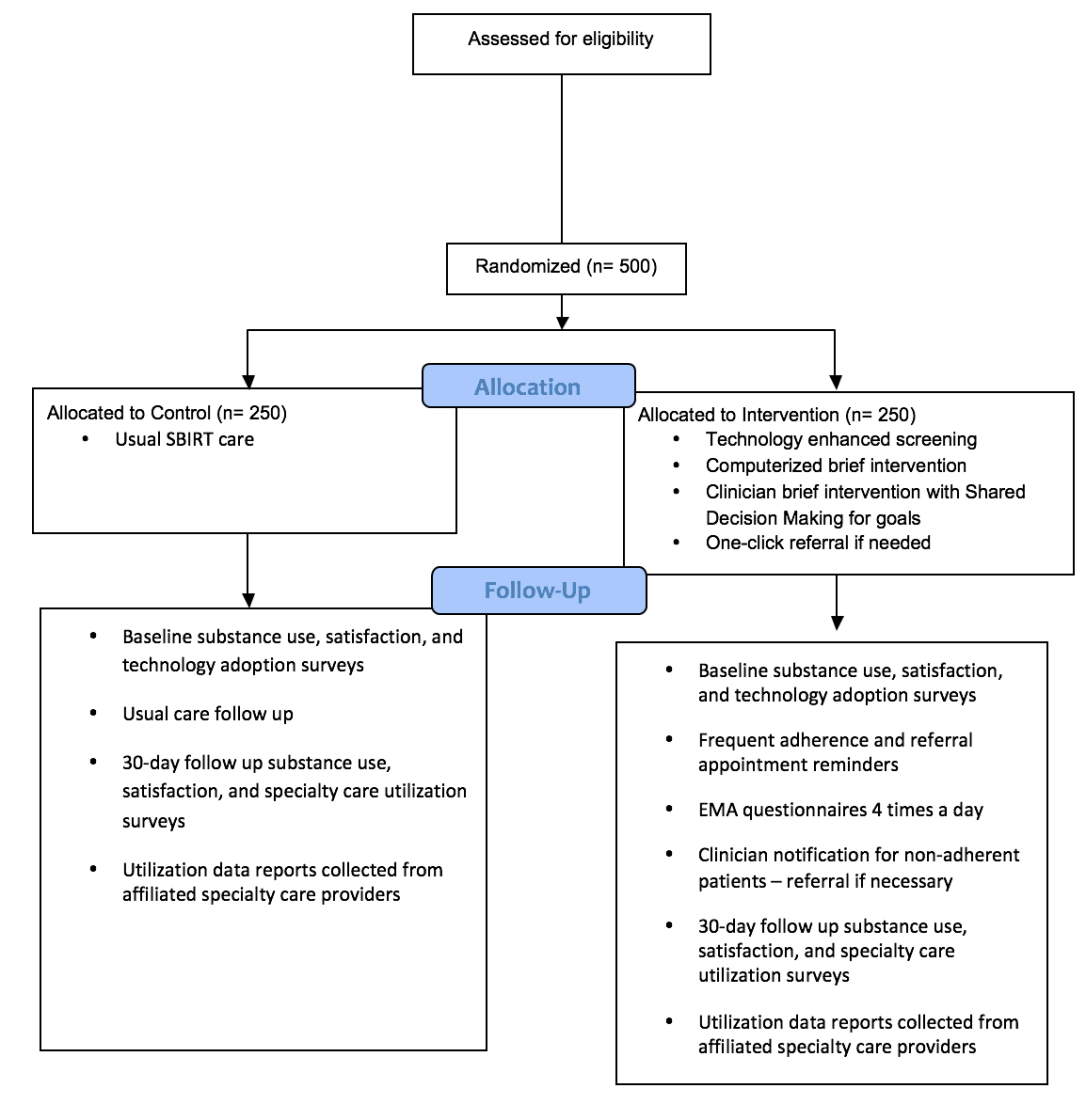
Study flow diagram.

Since the clinical trial involves more than 9 human subjects, the RCT will be approved by the OMB as well as the university’s Institutional Review Board (IRB), which is responsible for research in the university affiliated health care providers. Figure 1 displays the flow of participants.

#### Recruitment and Consent

RCT participants will be recruited from patients visiting the UHD Clinic for their regularly scheduled primary care visit. There will be a flyer in the reception area advertising the study. If a patient expresses interest in the study, a study coordinator will come out to greet the patient and lead him or her into a separate waiting room. The study coordinator will speak with the patient and go over the eligibility criteria (see Textbox 1).

**Eligibility criteria for study.**

Inclusion criteria:

- Aged 21 years and older
- University Health District Clinic patient
- Answered positively to single question drug use screener: “In the past year, have you used an illegal drug (including marijuana) or used a prescription medication for nonmedical reasons?”
- Willing to use personal mobile device for 30-day follow-up
- Consents to share personal data from specialty and primary care providers

Exclusion criteria:

- In the clinic for urgent conditions
- Cannot read or comprehend English at 6th grade level

Once the study coordinator decides that the patient is eligible to participate in the study, they will go over the informed consent together. The informed consent is approved by both OMB and the university’s IRB. The patient will sign the form and receive a copy of the signed document. Once the patient consents, he or she becomes a study participant.

#### Randomization

The study coordinator will then use an online random number generator (randomizer.org) to randomize the participant to either the control or intervention group.

The study administrator will then search for the patient name in the SBIRT application’s admin console. The admin console searches the connected electronic medical record (EMR) records and retrieves the patient data to populate the study’s database. The patient participant’s arm will be noted in the database record.

#### Screening

**Figure 2:**
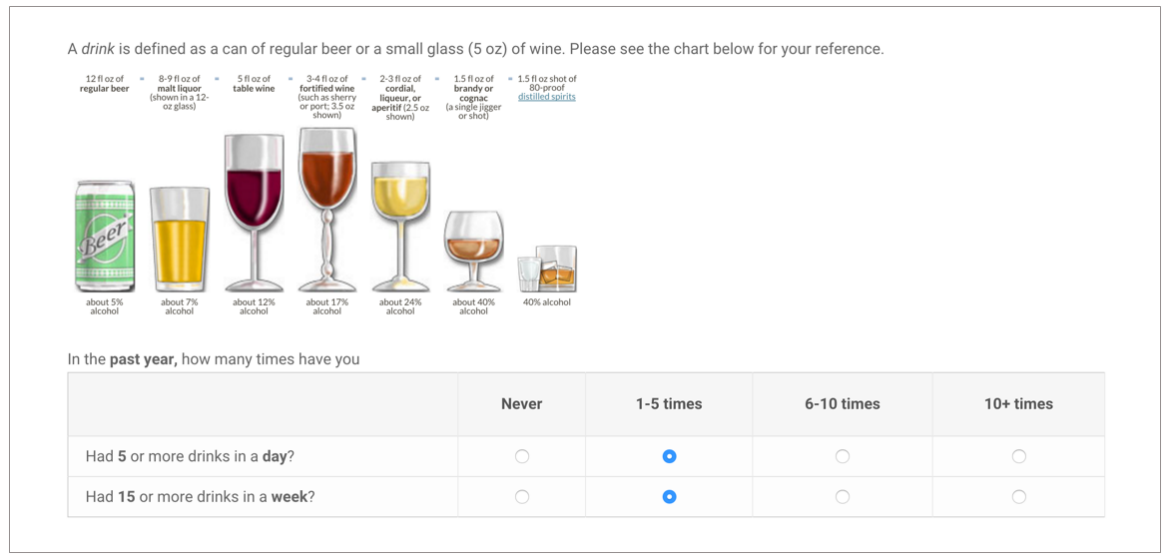
Dynamic branching screening tool, participant view.

In this study, we will use a standard set of screening instruments for participants in both arms. Control arm participants will be screened using paper questionnaires. Intervention arm participants will be screened using the dynamically branching iPad mobile app (see Figure 2). Although our recruitment screener only asks about drug use, we will screen for tobacco, alcohol, and drug use in this study, as many drug users have tobacco and alcohol problems that potentially need counseling or treatment. The following screening instruments will be used:

- One question screeners for tobacco, e-cigarette, alcohol, and drug use
- My Own Health Record questionnaire for diet, exercise, and lifestyle
- Patient Health Questionnaire (PHQ-9) for depression screening
- Alcohol Use Disorders Identification Test (AUDIT-C) questionnaire for patients positive for the 1-question alcohol screener
- DAST-10 questionnaire for patients positive for the 1-question drug use screener

#### Brief Intervention

For control arm participants, usual care will be provided. Screening scores will be manually computed and entered into the EMR by a clinician trained in SBIRT. Clinician will deliver brief intervention to participants who score moderate to high risk in any substance without technology-enhanced intervention.

For intervention arm participants, the brief intervention in the primary care office will consist of 4 steps.

Participant will be asked to rate his or her willingness to quit for all his or her at-risk substances on the iPad app.

**Figure 3:**
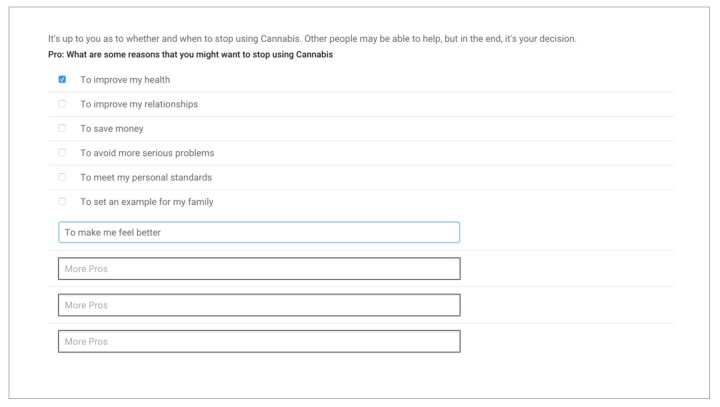
Brief intervention, participant view.

For the substance he or she is most likely to quit, the participant completes a brief 2-part intervention using an iPad app: watching a 3-minute educational video about the substance and its risks and completing an exercise to name the pros and cons of quitting this substance (Figure 3).

**Figure 4:**
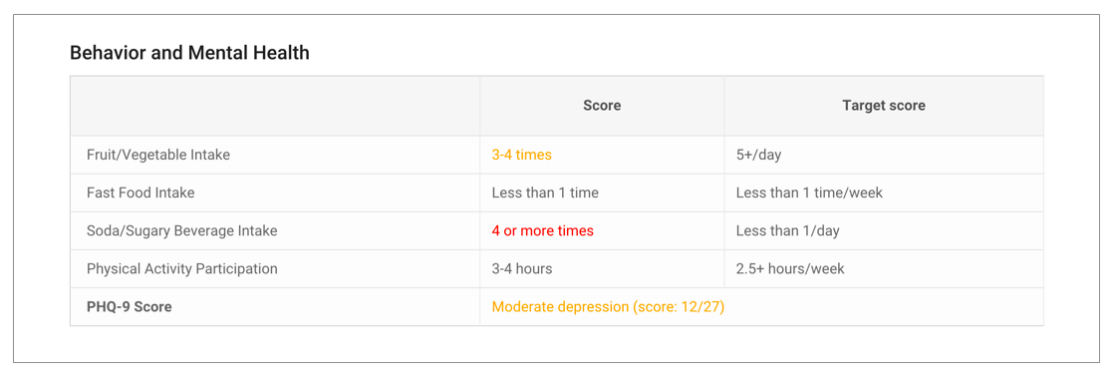
Brief intervention, clinician view.

When the clinician comes into the room, the participant hands the iPad over to the clinician. The clinician now has an overview of screening results, readiness to change for at-risk substances, and the participant’s personal pros and cons for the change. The clinician will conduct the brief intervention in person (Figure 4).

**Figure 5:**
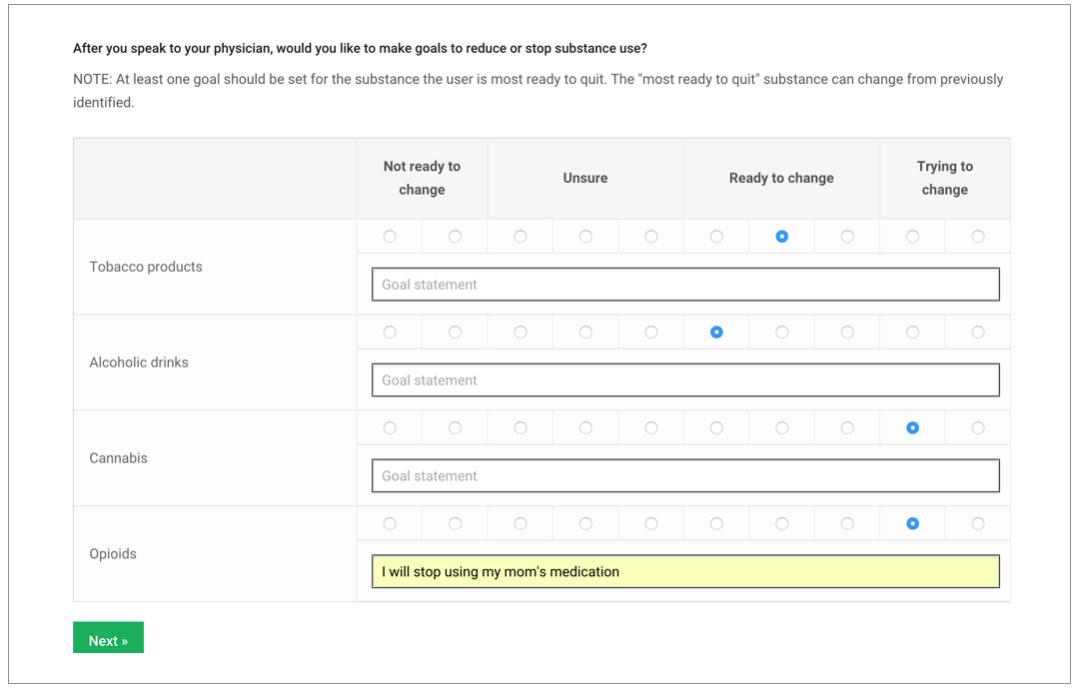
Setting goals, participant view.

At the end of the brief intervention, the clinician will ask the participant to rate readiness for change again. They will work together to set up quitting or reducing use goals for at least one at-risk substance. At the end, the participant will sign on the screen and commit to the goals (Figure 5).

#### Referral to Treatment

If the participant is screened at high risk for any substance and the clinician and participant mutually agreed to specialty follow-up treatment, the clinician can easily refer the participant to a specialty treatment program affiliated with this study from the iPad app. The participant referral data is also integrated into EMR.

#### Follow-Up

The control arm participants will receive no additional follow-up beyond usual care until the 30 days’ end.

The intervention arm participants will receive a once-per-day reminder to answer daily follow-up questions. The questionnaire screen includes:

- Reminder for specialty care appointment the day before the appointment. The user can reschedule the appointment from the questionnaire screen. The questionnaire also asks whether the user went to the appointment on the day after.
- Adherence by participant to each of his or her own stated goals on that day.
- Using items from a drug use self-monitoring log developed by Sobell and Sobell [16], participants will be asked to report details of their drug and alcohol use within the last 24 hours, including drugs used, drinks had, cravings or urges to use drugs or drink alcohol, situations related to drug use (eg, patient was alone, in a social situation), and thoughts or feelings experienced when using drugs or during urges.

In the event of strong craving or substance use, the user is instructed to access an EMA screen bookmarked on his or her phone. The event-based EMA measures craving and surroundings at the time of craving or substance use.

- Adapted from the 3-item Opioid Craving Scale by McHugh et al [31], participants will be asked to rate on a scale from 1 to 10 how much they currently crave a drug, how strong their desire to use has been when something in the environment reminded them of the drug, and the likelihood that they would use the drug in a specific environment.
- EMA items on psychological mood are adapted from an EMA assessment conducted by Gwaltney et al [32] and are designed to assess the participant’s affect state associated with substance use. The items ask participants to rate to what extent they feel happy, stressed, relaxed, bored, irritable, energized, and sleepy.

A risk score will be computed for every intervention participant every day. The score depends on the participant’s questionnaire adherence, self reported goal adherence, and substance use. If a participant is at high risk, he or she may be offered the brief intervention video to watch again.

**Figure 6:**
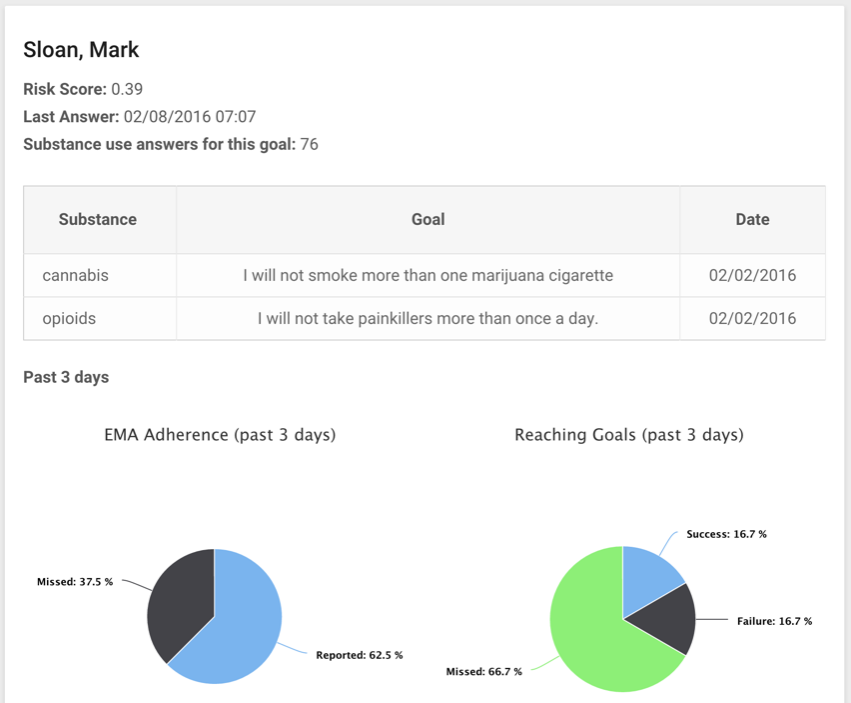
Web-based dashboard for intervention or additional referral, clinician view.

The risk score and individual participant summaries will be made available to the participant’s clinicians, including a primary care physician and the study coordinator who also serves as a substance use coach, via a Web-based dashboard (Figure 6). If the clinicians determine that the participant’s follow-up pattern or answers indicate high-risk substance use behavior, they may make additional referrals for the participant.

#### Surveys

At the end of the primary care clinic visit, all participants will be asked to complete a survey about their experience with the process and their knowledge about SUDs. The intervention group patients will also be asked about their acceptance of the technology solution. The survey has the following components:

- A 19-item validated computer system usability questionnaire is used to evaluate a computer system’s usability [33]. It also allows users to give free form answers on what he or she likes or dislikes about the system. We use it to evaluate the iPad tool in primary care clinics.
- A questionnaire based on the unified theory of acceptance and use of technology (UTAUT) by Venkatesh et al [34] is used to evaluate the technology solution’s ease-of-use, perceived usefulness, and the user’s social and facilitating conditions to predict the adoption of such technology solutions. The original UTAUT questions will be adapted to reflect our product.
- A 10-item validated patient satisfaction questionnaire will evaluate patient satisfaction with primary care providers [35].
- The ASI–Lite asks for drug use in the previous month, comorbid medical and mental illness, and social and legal outcomes related to drug use [36]. The ASI score is a quantitative measure of the user’s substance use.

At the end of the 30-day follow-up period, all participants will be asked to complete the screening, patient satisfaction, and ASI questionnaires again. They will also be asked to report any health care service they received during the 30 days, including any specialty SUD services. They can complete those questions on a computer or on paper. A complete schedule of all participant surveys and questionnaires is listed in Table 1.

#### Clinician Interviews

At the end of the study, all participating clinicians will be interviewed and debriefed on a one-to-one basis. Some clinicians will have worked on both arms of participants and can provide valuable insights on where the technology solution succeeded and where it still needs improvements. Clinician acceptance is a critical factor for successful commercialization and dissemination of the technology intervention in the future.

**Table 1:** Schedule of survey and questionnaire completion by study arm.

	Intervention arm				Control arm		
	Screening	End of PCP^a^ visit	Follow-up	30 days	Screening	End of PCP visit	30 days
One question screener	x				x		
My Own Health Record	x				x		
PHQ-9^b^	x				x		
AUDIT-C^c^	Optional			x	x		x
DAST-10^d^	Optional			x	x		x
Change readiness		x		x	x		x
Technology acceptance (UTAUT^e^)		x					
Computer system usability		x					
Patient satisfaction		x		x		x	x
Addiction Severity Index–Lite		x		x		x	x
Goal adherence			Daily				
Psychological mood			EMA^f^				
Context/surroundings			EMA				
Substance use			Daily				
Substance craving scale			EMA				
Referral adherence			Optional				
Specialty care utilization				x			x

^a^PCP: primary care provider.

^b^PHQ-9: Patient Health Questionnaire.

^c^AUDIT-C: Alcohol Use Disorders Identification Test.

^d^DAST-10: Drug Abuse Screening Test.

^e^UTAUT: unified theory of acceptance and use of technology.

^f^EMA: ecological momentary assessment.

### Data Collection and Analysis

#### Data Sources

In this RCT, we collect data from 2 primary sources: the participant self-reported data through surveys and questionnaires and clinic-reported utilization from reports.

#### Surveys and Questionnaires

Each participant will complete multiple surveys and questionnaires at various points during this study (see Table 1).

#### Hypotheses Testing

Since the hypotheses are primarily concerned about the observed differences between control and intervention arms, including referral rates, satisfaction level, knowledge level, and greater reduction in substance use, Student *t* tests will be used.

A chi-square test will be performed on the proportions of participants who receive specialty care to detect the significance in the difference between control and intervention arms. This test will be repeated for participants who have self-reported receiving specialty care. A chi-square test will be performed on the proportions of participants who receive specialty care referral at the end of the primary care encounter and who receive a specialty care referral during the 30-day follow-up period. For each question in the participant satisfaction survey and on the overall computed satisfaction and knowledge score, we will perform a *t* test for the answer’s mean value. Clinician interview results will be evaluated qualitatively to determine clinicians’ satisfaction with the solution.

Difference in differences regression tests will be performed on the differences in self-reported substance use amount and frequency and DAST-10 scores measured at baseline and 30 days of the study. The correlation coefficients and their confidence levels will be computed. These tests detect difference in drug use changes from control and intervention groups during the intervention period.

#### Exploratory Analysis

The exploratory analysis will be performed to gain further insights into additional data collected from the intervention participants. We will perform linear regression analysis to associate model technology acceptance factors with the participant’s inclination to adopt the technology in the future. This result will inform us on which factors are most important to patients for this tool. We will perform linear regression analysis to associate observed follow-up metrics such as ratio of missed questionnaires, substance use, and substance craving with participant referral decisions during the follow-up period. The result will inform a model to predict high-priority patients for referral in future interventions.

The association between mood and substance use and craving is inconclusive in existing research literature. In this project, we will collect self-reported mood and substance use/craving data from all intervention arm participants. We will group substance use/craving data into positive and negative mood categories and compare the differences using chi-square or *t* tests of the mean methods as appropriate. Furthermore, we can apply multilevel (also called mixed effect) regression models to EMA data. Mixed effects regression models can be applied to normally distributed continuous and categorical outcomes and nonnormally distributed outcomes such as counts with a Poisson distribution. Mixed effects models are also robust to missing data because time is treated as a continuous variable, the implication being that subjects are not assumed to have the same number of assessments at the same time points. To evaluate the relationship between craving, mood, and substance use, we used a generalized linear mixed model with a binary logistic response function of substance use. Fixed effects included craving for the substance and mood states.

#### Power Calculation

For primary hypothesis 1, we will perform a chi-square test to evaluate the difference in proportions of participants who receive specialty care in the 2 arms. We estimate that SBIRT intervention [37] could result in 10% to 30% of patients receiving specialty care. We computed power using the following assumptions: chi-square for 2 proportions, 2 samples, and 2-sided test; type 1 error rate of 5%; power of 0.9; control arm with 20% participants receiving specialty care; intervention arm with 35% participants receiving specialty care; and same number of participants in control and intervention. A sample of 197 participants in each of arms will be sufficiently powered to detect such differences between control and intervention arms.

For primary hypothesis 2, we assume that both arms start with the same levels of substance use. Therefore, to test the hypothesis, we will test the mean substance use levels at 30 days for both arms. We computed power for the sample *t* test of the mean using the following assumptions: 2-tailed *t* test for the mean of 2 samples; type 1 error rate of 5%; power of 0.9; medium Cohen effect size; and same number of participants in control and intervention. A sample of 86 participants in each arm will produce sufficient power. Based on the above calculation and considering up to 20% attrition, a sample of 500 total participants will have sufficient power to test both primary hypotheses.

## Results

In phase 1 of this project, we developed and piloted a prototype solution to enhance SBIRT in primary care office and follow up patients intensively for 30 days. The prototype proved feasibility of the technology, including the innovative user interface, dynamic screening logic, computerized brief intervention, shared-screen SDM, and bidirectional EMR connectivity. Our pilot clinicians and patients overwhelmingly determined the tool to be easy to use and not intrusive to normal clinical workflows. Patients with low-risk SUDs were also able to adhere to the shared goals during the follow-up period.

In phase 2 of this project, we supposed that, compared with the control group participants at 30 days, (1) significantly more intervention group patients will receive specialty SUD care and (2) intervention group patients will report a greater reduction in substance use and a greater drop in DAST-10 scores. Our secondary hypotheses include that intervention group patients receive more referrals at the primary care clinic and during 30 days of follow-up, and both clinicians and patients are more satisfied with the technology-enhanced solution.

Data collection for phase 2 is well underway. We expect to report the data analysis results in early 2018.

## Discussion

### Summary

In this project, we propose to demonstrate a technology-enhanced SBIRT process that builds on SDM principles. Our solution provides automated implementation of validated screening measures in an easy-to-use mobile device–based screening tool to be used inside the primary care office. Since it is fully integrated in the clinicians’ workflow, the solution improves reliability and efficiency and provides automated EMR documentation. The ease of use and documentation could increase SBIRT-related reimbursement and could increase the number of patients screened for SUDs in primary care settings.

Evaluating change readiness and setting goals are key elements of all cognitive behavioral therapy–based interventions [3]. SDM showed significant improvements with regard to drug use behavior or depression compared with standard decision-making processes [15,38]. Using digital technologies on a shared mobile device inside a primary care office could improve the collaboration between clinician team and the patient, making it easier to use SDM in practice.

Our solution provides ready access to standardized preferred referral resources to use with patients during the brief intervention and/or referral to treatment stages of the process. The primary care provider can easily identify the appropriate referral target, consult with the patient, and complete the referral on the spot. By integrating the primary care and specialty care EMR systems, our system enables providers to follow-up with at-risk patients and potentially create risk profiles for nonadherent patients for early intervention.

Traditional SBIRT practice happens during the clinical encounter, but evidence suggests that following up with patients at home and repeated intervention will improve patient outcomes [39]. Furthermore, the follow-up period provides additional opportunities to identify at-risk patients for referrals if the patient fails to adhere to the shared goals.

### Conclusion

A key objective of this project is an expected deliverable of the National Institute on Drug Abuse: “Demonstrate efficacy to increase significantly the proportion of primary care patients who are successfully linked to and receive indicated follow-up specialty SUD care.” This research project seeks to enhance the standard practice of SBIRT through a mobile solution easily incorporated into primary care that will promote SDM and increase referral and adherence to specialty care through continued follow-up care. By conducting an OMB-approved RCT in primary care and SUD specialty service providers, we seek to prove that the enhanced digital SBIRT approach will increase referral from primary care, reduce substance use in the intervention population, and improve SUD patient outcomes compared to the control group.

## Abbreviations

ASI: Addiction Severity Index
AUDIT-C: Alcohol Use Disorders Identification Test
DAST-10: Drug Abuse Screening Test
EMA: ecological momentary assessment
EMR: electronic medical record
IRB: Institutional Review Board
OMB: Office of Management and Budget
PHQ-9: Patient Health Questionnaire
RCT: randomized controlled trial
SUD: substance use disorder
SBIRT: Screening, Brief Intervention, and Referral to Treatment
SDM: shared decision making
UHD: university health district
USPSTF: US Preventive Services Task Force
UTAUT: unified theory of acceptance and use of technology
